# Loneliness and its association with psychological and somatic health problems among Czech, Russian and U.S. adolescents

**DOI:** 10.1186/s12888-016-0829-2

**Published:** 2016-05-04

**Authors:** Andrew Stickley, Ai Koyanagi, Roman Koposov, Marek Blatný, Michal Hrdlička, Mary Schwab-Stone, Vladislav Ruchkin

**Affiliations:** Stockholm Centre for Health and Social Change (SCOHOST), Södertörn University, 141 89 Huddinge, Sweden; Department of Child and Adolescent Mental Health, National Institute of Mental Health, National Center of Neurology and Psychiatry, Kodaira, Tokyo Japan; Department of Human Ecology, Graduate School of Medicine, The University of Tokyo, Tokyo, Japan; Research and Development Unit, Parc Sanitari Sant Joan de Déu, Fundació Sant Joan de Déu, Sant Boi de Llobregat, Barcelona, Spain; Instituto de Salud Carlos III, Centro de Investigación Biomédica en Red de Salud Mental, CIBERSAM, Madrid, Spain; Regional Centre for Child and Youth Mental Health and Child Welfare, UiT The Arctic University of Norway, Tromsø, Norway; Institute of Psychology of the Czech Academy of Sciences, Veveří 97, 602 00 Brno, Czech Republic; Department of Child Psychiatry, Charles University Second Faculty of Medicine, University Hospital Motol, V Uvalu 84, 15006 Prague, Czech Republic; Child Study Centre, Yale University Medical School, New Haven, CT 06520 USA; Department of Child and Adolescent Psychiatry, Division of Neuroscience, Uppsala University, Uppsala, S-751 85 Sweden

**Keywords:** Loneliness, Adolescent, Correlates, Depressive symptoms, Somatic symptoms

## Abstract

**Background:**

Loneliness is common in adolescence and has been linked to various negative outcomes. Until now, however, there has been little cross-country research on this phenomenon. The aim of the present study was to examine which factors are associated with adolescent loneliness in three countries that differ historically and culturally-the Czech Republic, Russia and the United States, and to determine whether adolescent loneliness is associated with poorer psychological and somatic health.

**Methods:**

Data from a school survey, the Social and Health Assessment (SAHA), were used to examine these relations among 2205 Czech, 1995 Russian, and 2050 U.S. male and female adolescents aged 13 to 15 years old. Logistic regression analysis was performed to examine if specific demographic, parenting, personal or school-based factors were linked to feeling lonely and whether lonely adolescents were more likely to report psychological (depression and anxiety) or somatic symptoms (e.g. headaches, pain).

**Results:**

Inconsistent parenting, shyness, and peer victimisation were associated with higher odds for loneliness in at least 4 of the 6 country- and sex-wise subgroups (i.e. Czech, Russian, U.S. boys and girls). Parental warmth was a protective factor against feeling lonely among Czech and U.S. girls. Adolescents who were lonely had higher odds for reporting headaches, anxiety and depressive symptoms across all subgroups. Loneliness was associated with other somatic symptoms in at least half of the adolescent subgroups.

**Conclusion:**

Loneliness is associated with worse adolescent health across countries. The finding that variables from different domains are important for loneliness highlights the necessity of interventions in different settings in order to reduce loneliness and its detrimental effects on adolescent health.

**Electronic supplementary material:**

The online version of this article (doi:10.1186/s12888-016-0829-2) contains supplementary material, which is available to authorized users.

## Background

Loneliness is the emotionally unpleasant state which arises from the perception of a lack of desired interpersonal relationships [1]. Adolescents are particularly vulnerable to feeling lonely [1–3] with the reported prevalence of frequent loneliness among adolescents being higher than 50 % in one study [2]. Adolescent loneliness has been linked to the social and developmental changes taking place during this period. In particular, a growing need for autonomy and desire to establish a separate adolescent identity that stretches beyond the immediate family environment is reflected in increasing separation from parents and attempts to establish new relations with peers in the wider social world [2]. However, disproportionate and unrealistic expectations, feelings of rejection, a failure to forge appropriate social roles, as well as the parental blocking of this drive for greater independence, can all result in feelings of loneliness at this time [2].

Although loneliness is a universal [1] and normative [4] phenomenon, the experience of loneliness can nevertheless, take very different forms. Chronic loneliness has been linked to negative affectivity [5], while a recent commentary has highlighted how loneliness can be extremely painful and cause severe anguish [6] to a point where its consequences can even be life-threatening [7]. Understanding which factors are associated with adolescent loneliness and how it affects well-being is essential for formulating effective interventions to deal with this phenomenon and its effects.

### Correlates of adolescent loneliness

Factors that have been previously linked to feeling lonely in adolescence include ‘personal characteristics’ [7] such as shyness, a lack of self-esteem, poor social skills [2] and having fewer close friends [8, 9], whereas an increased number of friends and better quality relations may act as a buffer against childhood loneliness [10]. The family environment has also been associated with adolescent loneliness. Specifically, lower parental education [11], marital disruption (living in one parent/step families) [12] and different parenting styles such as inconsistent parenting [13] and parenting with high levels of warmth and involvement [14–17] have all been related to differences in adolescent loneliness. The school environment may also be important, as less interest in school activities, lower educational ambitions [2] and negative school attitudes [18] have also been previously associated with adolescent loneliness. Other adolescent school-based experiences might also affect loneliness as children who are victimised by peers are more likely to report feelings of loneliness [19, 20].

### Loneliness and adolescent health

There has been comparatively little research on the relation between loneliness and health outcomes among adolescents. Some studies have nonetheless highlighted a possible link between loneliness and depressive symptoms in adolescence [21–23]. Other adolescent health outcomes, such as somatic symptoms have however, been little explored [24]. The few studies which exist have indicated that adolescent loneliness may be associated with somatic symptoms such as headaches [24], anxiety, and gastrointestinal symptoms [25], and with detrimental symptom patterns (i.e. ‘psychological, physical, and psychosomatic manifestations of psychological distress’) more generally [26].

### Current study

To date, most of the research conducted on adolescent loneliness has been undertaken in the West, especially in North America [27], and there have been relatively few cross-country studies. In response to this, the current study will examine loneliness and its effects on health among adolescents in three countries-the Czech Republic, Russia and the United States. These countries were part of a large international research project focusing on adolescent well-being and its correlates and have different cultures and histories which might be impacting on loneliness and its effects, given that previous research has indicated for example, that culture is important when it comes to understanding the causes of loneliness [28].

Given the near total absence of research on adolescent loneliness and its effects in Eastern Europe, and the lack of comparative data from multi-country studies, the current study had two main aims: (1) to determine the factors associated with loneliness among Czech, Russian and U.S. adolescents and whether these vary across the three countries; and (2) to examine the degree to which loneliness affects psychological and somatic health among adolescents in the three countries.

## Methods

### Participants and procedure

Data in this study came from the Social and Health Assessment (SAHA) conducted in the Czech Republic, Russia and the U.S. in 2003. The primary aim of this survey was to determine the factors associated with adolescent health and well-being. The study sites were the following: Russia [the city of Arkhangelsk (population 360,000)]; the U.S. [the city of New Haven, Connecticut (population 125,000)]; and the Czech Republic [the capital Prague (population 1.2 million) and all 12 regional capitals (population 50,000–400,000)]. In Arkhangelsk and the Czech study locations, data were collected from a representative sample of students aged 12–17 and 12–16, respectively, in the cities’ public schools. In New Haven, all students aged 13–17 who were in the public school system were included. Students were recruited from within classes that were randomly selected from within schools that had themselves been randomly selected (excepting New Haven, where all students were included). In all countries, students completed the survey in their classrooms during a normal school day. Written informed consent was obtained from all participants prior to the survey administration, and both parents (for their children) and children had the right to refuse to participate. Response rates for these surveys were high with only 3.6 % of children refusing to participate in Russia, 1.4 % in the Czech Republic and <1 % in the United States. For comparability, the present study is limited to those adolescents who were aged 13–15 years old with the analytical sample thus comprising 2205 adolescents from the Czech Republic, 1995 from Russia and 2050 from the United States.

### Measures

*Loneliness* was measured using a question taken from a modified version of the Centre for Epidemiologic Studies-Depression Scale (CES-D) [29]. This instrument has been validated in the Czech Republic [30], while a previous study in Russia has similarly reported good psychometric properties for the instrument [31]. Students were asked to think about how they ‘felt or behaved in the past 30 days’. In response to the statement ‘I felt lonely’, students were presented with three response options: ‘Not true’, ‘Somewhat true’, and ‘Certainly true’. In a desire to examine more severe manifestations of loneliness, the ‘Not true’/‘Somewhat true’ answers were combined as the reference category (scored ‘0’) while ‘Certainly true’ was taken as signifying the most intense feeling of loneliness (scored ‘1’).

Information on *personal characteristics* was obtained by asking students to respond to the statement ‘I am shy’ where the response options were ‘Not true’, ‘Somewhat true’, and ‘Certainly true’. *Friendship ties* were assessed by asking students to indicate the number of close friends they had, with response options ranging from ‘0’ to ‘5 or more’. This variable was dichotomised into having 0 and ≥1 friend.

Details of the family environment were obtained through three measures. *Parental education* was used as a marker of the family’s socioeconomic status. This variable was categorised into ‘graduated from college’ (‘high education’) and having less than a college graduate’s education (‘low education’). If both parents (or the male or female guardian) were present in the home, the highest educational level was used as the category for that family. As a large number of cases were missing for this variable (the Czech Republic 10.5 %, Russia 22.0 %, the U.S. 16.8 %,), a third category, ‘missing’, was also created to prevent these subjects from being excluded from the analysis. *Family structure* was assessed as being either ‘intact’ when both biological parents were present, ‘restructured’ where a biological parent and step parent were together, ‘single parent’ where there was one biological parent and no step parent and ‘other’ for any other form of arrangement where neither biological parent was present. Students were also asked how many people (including themselves) lived in their home. We categorised this *household size* variable into ‘2’ persons and ‘≥3’ persons.

Information was also obtained about the perceptions of parental behaviour using three variables that came from Parenting Scales developed by the SAHA Research Evaluation Team [32]. All of them used the same scoring system for individual questions [‘Never’ (scored ‘1’), ‘Rarely’ (scored ‘2’), ‘Sometimes’ (scored ‘3’) and ‘Often’ (scored ‘4’)] and a composite score was created by adding the scores of all the individual questions used for that variable. The *inconsistent parenting* scale consisted of five items that asked about inconsistent parenting practices using statements such as ‘My parents or guardians…only keep rules when it suits them’. Scores ranged from 5 to 20 with higher scores indicating more inconsistent parenting (Cronbach’s α = 0.65). The *parental involvement* scale consisted of six items assessing youth perceptions of the degree to which their parents and/or primary guardians were involved and interested in their lives using statements such as ‘My parents…ask me about my life’. Scores ranged between 6 and 24 with higher scores indicating greater parental involvement (α = 0.73). Finally, the *parental warmth* scale consisted of 5 statements on adolescents’ perceptions of their parents’ warmth and support for them using statements such as ‘My parents…show their love for me’. Scores ranged from 5 to 20 with higher scores indicating greater parental warmth (α = 0.81).

Two school-based factors were examined. *School attachment* was assessed using the statement ‘I like school’. Answers options to this question were ‘Definitely not true’, ‘Mostly not true’, ‘Mostly true’ and ‘Definitely true’. This variable was dichotomised into Definitely/Mostly not true (0) vs. Definitely/Mostly true (1). School-based *peer victimisation* was assessed by asking about the experience of victimisation using an adapted version of the Multidimensional Peer-Victimisation Scale [33] which included questions on four main types of school-based peer victimisation-physical victimisation, social manipulation, verbal victimisation and attacks on property. Using nine statement items such as ‘During this school year other kids in school…called me names or swore at me’, the responses to each statement item, which referred to the frequency of the behaviour [‘Not at all’ (scored ‘0’), ‘Once’ (scored ‘1’), ‘2-3 times’ (scored 2) and ‘4 or more times’ (scored ‘3’)], were summed to create a scale ranging from 0 to 27 where higher scores indicated greater victimisation (α = 0.83).

#### Psychological and somatic health variables

We examined the relation between feeling lonely and different psychological and somatic symptoms using items taken from the Social and Health Assessment (SAHA) survey instrument [32]. Despite the close links between loneliness and depression, previous studies have treated these psychological variables as separate constructs [5] and several studies have focused on the relation between loneliness and depression in adolescence [23, 34]. We assessed *depressive symptoms* using a composite score based on a modified version of the CES-D [29] after removing a question on loneliness-a method which has been used in previous studies [35, 36]. The scale consisted of nine items where response options were ‘Not true’ (scored ‘0’), ‘Somewhat true’ (scored ‘1’), and ‘Certainly true’ (scored ‘2’). Item answers were summed to form a scale score ranging from 0 to 18 (α = 0.84). We assessed *anxiety symptoms* using a scale that consisted of 12 statements with the same answer options as for depressive symptoms. This created a scale that was scored from 0 to 24 with higher scores indicating greater anxiety (α = 0.85). As the scores on the depressive and anxiety symptoms varied between countries, they were examined in two ways. We used an overall top-quintile cut-off point across the three countries, and also country-specific top-quintile cut-off scores. Seven types of *somatic symptoms* were assessed based on symptoms in the past 30 days. Answer options were dichotomised into either not experiencing that symptom [i.e. ‘Not true’ (scored ‘0’)] or experiencing it [i.e. ‘Somewhat true’, ‘Certainly true’ (scored ‘1’)].

### Additional files

The survey questions that were used in this study are presented in Additional file 1.

### Statistical analysis

As previous research has shown that the prevalence of loneliness and other factors related to loneliness vary by sex among adolescents in some of these countries [36], we stratified the analysis by sex. We used univariable and multivariable logistic regression analyses to assess which factors were associated with adolescent loneliness, and the relation between loneliness and the health outcomes (i.e. depressive, anxiety, and somatic symptoms). In the multivariable analysis that assessed which factors were associated with loneliness, all of the covariates presented in the models in Table 2 were mutually adjusted. When assessing the association between loneliness and the health outcomes, the multivariable analysis was adjusted for age, family structure, parental education, and peer victimisation. The selection of the covariates to be included in the analysis was based on past literature. Parental warmth, involvement, inconsistent parenting, and peer victimisation were included in the analysis as continuous variables. As the results were similar between the univariable and multivariable analyses, for the sake of brevity, we only present the results from the multivariable analysis in the tables. The results are presented as odds ratios (OR) with 95 % confidence intervals (CI). Clustering within schools was adjusted for by using the clustered sandwich estimator. The analyses were performed using the statistical software package Stata 12.1 (Stata Corp LP, College Station, Texas) with *p* <0.05 taken as signifying statistical significance.

### Ethical considerations

Ethical approval for the study was obtained from ethical committees at the Northern State Medical University in Arkhangelsk, Yale University School of Medicine and the Institute of Psychology, Academy of Sciences of the Czech Republic, v.v.i.

## Results

### Descriptive statistics

In every country, the prevalence of loneliness was higher among girls than boys: U.S. (14.7 vs. 6.7 %); Czech Republic (10.6 vs. 5.2 %); and Russia (14.4 vs. 8.9 %). The characteristics of the study sample are presented in Table 1. The U.S. sample was younger, with fewer intact families and with a lower level of parental education compared to in the other countries.

**Table 1:** Characteristics of the study sample

		Czech Republic		Russia		U.S.	
Characteristic	Categories	Female	Male	Female	Male	Female	Male
Demographic							
Age (years)	13	28.4 (321)	33.5 (359)	13.6 (150)	14.3 (127)	59.7 (611)	53.6 (550)
	14	43.1 (488)	35.6 (382)	41.8 (462)	44.8 (399)	34.9 (357)	37.1 (381)
	15	28.5 (323)	30.9 (332)	44.6 (493)	40.9 (364)	5.5 (56)	9.3 (95)
Family structure	Intact	70.7 (800)	70.7 (759)	66.4 (730)	64.3 (571)	31.3 (320)	36.2 (371)
	Restructured	11.1 (126)	9.5 (102)	5.8 (64)	5.5 (49)	18.1 (185)	17.5 (179)
	Single parent	16.5 (187)	17.2 (185)	24.2 (266)	24.9 (221)	39.6 (405)	36.4 (373)
	Other	1.7 (19)	2.5 (27)	3.6 (40)	5.3 (47)	11.1 (114)	10.0 (103)
Parental education	Low	43.8 (496)	42.1 (452)	29.9 (330)	31.2 (278)	54.0 (553)	51.9 (532)
	High	46.4 (525)	46.6 (500)	48.7 (538)	46.1 (410)	29.4 (301)	31.2 (320)
	Missing	9.8 (111)	11.3 (121)	21.5 (237)	22.7 (202)	16.6 (170)	17.0 (174)
Household size	2	5.5 (62)	6.0 (64)	10.6 (114)	8.6 (73)	5.7 (58)	5.3 (53)
	≥3	94.5 (1062)	94.0 (1004)	89.4 (963)	91.5 (781)	94.3 (953)	94.7 (952)
Parenting							
Parental warmth^a^	Mean (SD); n	16.6 (3.1); 1107	15.7 (3.1); 1032	16.2 (3.3); 1021	15.5 (3.2); 792	17.0 (3.5); 960	16.6 (3.5); 908
Parental involvement^a^	Mean (SD); n	17.2 (3.4); 1102	16.6 (3.3); 1041	17.6 (3.6); 1010	16.9 (3.6); 753	17.9 (4.1); 937	17.0 (4.1); 891
Inconsistent parenting^a^	Mean (SD); n	11.8 (3.4); 1101	11.8 (3.3); 1044	12.1 (3.3); 1022	11.8 (3.5); 803	12.6 (3.7); 902	12.4 (3.5); 881
Friendship ties							
Number of close friends	0	1.3 (15)	1.8 (19)	3.3 (36)	1.9 (17)	1.9 (19)	2.6 (26)
	≥1	98.7 (1104)	98.2 (1042)	96.7 (1048)	98.1 (862)	98.1 (991)	97.5 (993)
Personal characteristics							
I am shy	Not True	47.7 (534)	57.3 (606)	39.4 (427)	38.7 (327)	39.7 (392)	56.0 (534)
	Somewhat true	38.2 (428)	36.2 (383)	43.5 (472)	47.6 (402)	37.0 (365)	30.0 (286)
	Certainly true	14.1 (158)	6.4 (68)	17.1 (186)	13.7 (116)	23.3 (230)	14.0 (133)
School-based factors							
School attachment (I like school)	Not true	47.5 (536)	61.1 (654)	27.3 (300)	40.5 (356)	33.2 (337)	40.6 (412)
	True	52.5 (593)	38.9 (417)	72.7 (800)	59.6 (524)	66.8 (677)	59.4 (603)
Peer victimisation^a^	Mean (SD); n	3.6 (3.9); 1093	3.9 (4.5); 1030	4.0 (4.3); 1042	5.3 (5.5); 803	5.1 (5.0); 958	5.6 (6.0); 913

Data are % (n) unless otherwise specified

^a^These variables were composite scores. Higher scores on the parental warmth, involvement and inconsistency scales refer to higher levels of involvement, warmth and inconsistency, respectively. Higher scores on the peer victimisation scale correspond to higher levels of victimisation (refer to text for details)

### Correlates of adolescent loneliness

The factors associated with feeling lonely are presented in Table 2. For the demographic variables, although age was not associated with feeling lonely in the Czech Republic, U.S. boys aged 14 years old had higher odds of feeling lonely than their 13 year old counterparts, while the same was observed for 15 year old girls in Russia. Czech boys (OR: 3.10, 95 % CI: 1.12–8.57) and Russian girls (OR: 2.63, 95 % CI: 1.31–5.27) living in family structures categorised as ‘other’ had higher odds for feeling lonely compared to adolescents living in intact families. Parental involvement was not related to loneliness, while higher levels of parental warmth were associated with lower levels of loneliness among Czech and U.S. females. Inconsistent parenting was associated with higher odds for feeling lonely among boys and girls in every country with the sole exception of Czech females. Having at least one close friend reduced the odds of feeling lonely among Czech females (OR: 0.17: 95 % CI: 0.05–0.62) and U.S. males (OR: 0.15, 95 % CI: 0.07–0.35) compared to having no close friends. Strong feelings of shyness were associated with higher odds for feeling lonely in all groups except U.S. females. Although school attachment was not linked to loneliness in any of the countries, peer victimisation increased the odds for loneliness among girls in every country and among U.S. boys.

**Table 2:** Factors associated with feeling lonely among adolescents in the Czech Republic, Russia and the United States

		Czech Republic		Russia		U.S.	
		Female	Male	Female	Male	Female	Male
Characteristic	Categories	Adj. OR^a^	Adj. OR^a^	Adj. OR^a^	Adj. OR^a^	Adj. OR^a^	Adj. OR^a^
Demographic							
Age (years)	13	1.00	1.00	1.00	1.00	1.00	1.00
	14	1.32 (0.77–2.26)	0.81 (0.35–1.87)	1.74 (0.89–3.41)	2.21 (0.81–6.06)	0.93 (0.65–1.35)	2.18 (1.24–3.83)^**^
	15	1.48 (0.86–2.56)	1.09 (0.50–2.36)	2.32 (1.20–4.49)^*^	2.32 (0.74–7.23)	1.15 (0.45–2.96)	1.79 (0.47–6.80)
Family structure	Intact	1.00	1.00	1.00	1.00	1.00	1.00
	Restructured	0.59 (0.27–1.29)	1.53 (0.55–4.26)	1.69 (0.89–3.22)	1.50 (0.42–5.31)	0.92 (0.39–2.14)	0.60 (0.24–1.53)
	Single parent	0.83 (0.42–1.66)	1.78 (0.75–4.19)	1.23 (0.69–2.20)	1.72 (1.00–2.97)	0.80 (0.50–1.28)	0.64 (0.37–1.10)
	Other	1.80 (0.50–6.41)	3.10 (1.12–8.57)^*^	2.63 (1.31–5.27)^**^	0.81 (0.12–5.35)	0.94 (0.40–2.18)	1.30 (0.45–3.69)
Parental education	High vs. Low	1.23 (0.80–1.89)	1.01 (0.50–2.05)	1.15 (0.69–1.91)	0.96 (0.43–2.13)	0.86 (0.50–1.46)	1.25 (0.43–3.67)
Household size	≥3 vs. 2	0.70 (0.28–1.73)	0.81 (0.26–2.54)	1.65 (0.73–3.69)	0.54 (0.23–1.28)	0.77 (0.31–1.89)	0.90 (0.26–3.16)
Parenting							
Parental warmth^b^		0.89 (0.81–0.98)^*^	0.91 (0.81–1.01)	0.96 (0.87–1.07)	0.99 (0.86–1.14)	0.88 (0.79–0.98)^*^	0.88 (0.76–1.03)
Parental involvement^b^		0.98 (0.91–1.06)	0.95 (0.86–1.05)	0.96 (0.91–1.01)	1.03 (0.91–1.17)	1.03 (0.93–1.14)	1.01 (0.89–1.16)
Inconsistent parenting^b^		1.03 (0.95–1.11)	1.08 (1.00–1.16)^*^	1.07 (1.03–1.12)^**^	1.14 (1.04–1.25)^**^	1.09 (1.04–1.15)^**^	1.13 (1.03–1.24)^*^
Friendship ties							
Number of close friends	≥1 vs. 0	0.17 (0.05–0.62)^**^	2.00 (0.34–11.77)	0.61 (0.18–2.03)	0.22 (0.04–1.26)	1.93 (0.22–16.98)	0.15 (0.07–0.35)^***^
Personal characteristics							
I am shy	Not true	1.00	1.00	1.00	1.00	1.00	1.00
	Somewhat true	1.53 (0.93–2.51)	1.66 (0.76–3.66)	1.25 (0.80–1.95)	1.08 (0.64–1.81)	0.81 (0.44–1.48)	2.00 (1.11–3.60)^**^
	Certainly true	4.32 (2.45–7.64)^***^	4.36 (1.79–10.60)^**^	1.98 (1.20–3.27)^**^	4.03 (2.08–7.80)^***^	1.36 (0.87–2.14)	7.37 (3.88–14.00)^***^
School-based factors							
School attachment (I like school)	True vs. Not true	1.02 (0.66–1.59)	0.90 (0.42–1.90)	1.02 (0.66–1.58)	0.70 (0.43–1.12)	0.98 (0.66–1.46)	0.54 (0.26–1.14)
Peer victimisation^b^		1.13 (1.07–1.19)^***^	1.05 (0.98–1.12)	1.10 (1.05–1.15)^***^	1.02 (0.97–1.09)	1.10 (1.07–1.14)^***^	1.06 (1.03–1.09)^***^

^*^ *P* < 0.05, ^**^ *P* < 0.01, ^***^ *P* < 0.001

^a^Mutually adjusted for all covariates in the model

^b^These variables were composite scores and were included in the regression analysis as continuous variables. Higher scores on the parental warmth, involvement and inconsistency scales refer to higher levels of involvement, warmth and inconsistency, respectively. Higher scores on the peer victimisation scale correspond to higher levels of victimisation (refer to text for details)

### Adolescent loneliness and psychological and somatic health

The association between feeling lonely and experiencing psychological and somatic symptoms is reported in Table 3. Loneliness was strongly associated with adolescent depressive symptoms in all of the countries using both the common and country-specific cut-off points. The odds were especially high among U.S. adolescents. Lonely adolescents also had higher odds for experiencing anxiety symptoms in all countries (apart from Russian females when the country-specific cut-off point was used), with odds ratios ranging from 1.63 (Russian males) to 5.49 (U.S. males) when using a common cross-country cut-off point. In terms of the somatic symptoms, the strongest relation between feeling lonely and reporting somatic health problems was observed among U.S. adolescents, where loneliness was linked to higher odds for every symptom among boys and girls. Loneliness was also associated with higher odds for every somatic symptom among Czech boys with the exception of visual problems. For Czech girls and Russian boys, feeling lonely was associated with increased odds for three of the seven somatic symptoms, while lonely Russian girls had higher odds for having headaches and feeling nauseous.

**Table 3:** Association between loneliness and psychological and somatic symptoms among Czech, Russian and U.S. adolescents

		Czech Republic		Russia		U.S.	
		Female	Male	Female	Male	Female	Male
Outcome	Loneliness	Adj. OR (95 %CI)^a^	Adj. OR (95 %CI)^a^	Adj. OR (95 %CI)^a^	Adj. OR (95 %CI)^a^	Adj. OR (95 %CI)^a^	Adj. OR (95 %CI)^a^
Psychological symptoms^b^							
Depressive symptoms	Yes vs. No	10.65 (6.84–16.56)^***^	24.86 (13.00–47.51)^***^	10.71 (7.43–15.44)^***^	14.37 (9.83–21.02)^***^	40.13 (19.49–82.59)^***^	22.53 (10.49–48.39)^***^
Depressive symptoms	Yes vs. No	8.04 (5.22–12.39)^***^	18.27 (9.62–34.70)^***^	13.51 (8.85–20.63)^***^	14.90 (10.41–21.32)^***^	40.13 (19.49–82.59)^***^	22.53 (10.49–48.39)^***^
(country-specific cut-off)							
Anxiety symptoms	Yes vs. No	2.98 (1.91–4.64)^***^	2.68 (1.30–5.51)^**^	1.66 (1.04–2.64)^*^	1.63 (1.07–2.48)^*^	2.98 (2.10–4.25)^***^	5.49 (3.41–8.82)^***^
Anxiety symptoms	Yes vs. No	3.38 (2.30–4.97)^***^	3.04 (1.65–5.61)^***^	1.30 (0.70–2.41)	2.01 (1.11–3.67)^*^	3.06 (2.33–4.03)^***^	6.17 (3.81–10.00)^***^
(country-specific cut-off)							
Somatic symptoms^c^							
I had headaches	Yes vs. No	2.20 (1.46–3.31)^***^	1.88 (1.07–3.31)^*^	1.53 (1.06–2.22)^*^	1.78 (1.21–2.63)^**^	2.55 (1.74–3.74)^***^	3.12 (2.20–4.43)^***^
I had stomach aches	Yes vs. No	1.73 (1.09–2.75)^*^	2.14 (1.19–3.85)^*^	1.09 (0.78–1.53)	1.18 (0.78–1.78)	2.61 (1.82–3.73)^***^	3.04 (2.09–4.43)^***^
I had aches or pains	Yes vs. No	1.34 (0.83–2.18)	1.94 (1.11–3.39)^*^	1.43 (0.99–2.07)	1.32 (0.83–2.12)	3.03 (1.98–4.66)^***^	5.25 (2.42–11.40)^***^
I had nausea	Yes vs. No	1.56 (0.97–2.49)	2.30 (1.16–4.56)^*^	1.49 (1.12–1.97)^**^	2.25 (1.54–3.27)^***^	3.70 (2.15–6.35)^***^	4.52 (2.18–9.37)^***^
I had problems with my eyes	Yes vs. No	2.02 (1.41–2.90)^***^	1.52 (0.84–2.73)	0.90 (0.73–1.12)	0.75 (0.34–1.66)	2.33 (1.45–3.74)^***^	2.62 (1.17–5.84)^*^
I had rashes/other skin problems	Yes vs. No	1.36 (0.88–2.11)	2.08 (1.01–4.28)^*^	1.18 (0.73–1.89)	1.58 (0.93–2.70)	1.46 (1.25–1.71)^***^	2.26 (1.07–4.76)^*^
I was vomiting	Yes vs. No	1.30 (0.71–2.37)	3.29 (1.52–7.09)^**^	0.96 (0.48–1.89)	2.58 (1.64–4.08)^***^	3.20 (2.32–4.40)^***^	3.47 (1.70–7.10)^**^

^*^ *P* < 0.05, ^**^ *P* < 0.01, ^***^ *P* < 0.001

^a^Adjusted for age, family structure, parental education, and peer victimisation

^b^Depressive and anxiety symptoms were defined as the overall or country-specific highest quintile of composite scores (refer to text for details). The overall and country-wise cut-off values for depressive symptoms were the same for the U.S

^c^Responses to somatic symptoms were dichotomised as not true (reference) and somewhat/certainly true

## Discussion

This study has shown that approximately one in ten Russian and U.S. adolescents, and one in thirteen Czech adolescents report strong feelings of loneliness, and that girls are more likely to report being lonely than boys in every country. Moreover, certain characteristics such as shyness, inconsistent parenting and experiencing peer victimisation are strongly related to adolescent loneliness in all of the countries, while other factors such as family structure, parental warmth and friendship ties are linked to loneliness among some groups in some of the countries. The study also revealed a close link between loneliness and poorer adolescent health in all of the countries, with an especially strong relation being seen among U.S. adolescents and Czech boys.

### Factors associated with adolescent loneliness

Previous studies have produced mixed findings concerning the importance of family structure for adolescent loneliness [12, 37–39]. In those studies where differences have been detected, children who live in non-intact families have had an increased risk of feeling lonely [12, 37], possibly because non-intact family households may have poorer relational networks and thus fail to provide the necessary degree of affection and support which might protect against loneliness [37]. Although caution should be exercised when discussing our findings as the numbers involved were small, the fact that the ‘other’ category was the only form of family structure linked to loneliness suggests that not living with at least one biological parent may be especially detrimental for adolescent well-being in terms of feeling lonely. It is possible that such family structures may have a low level of family cohesion, which has previously been identified as a risk factor for loneliness in early adolescence [40].

The parenting variables had differing effects in terms of loneliness: parental involvement was not associated with feeling lonely in any of the countries, greater parental warmth was linked to a reduced risk for females in the Czech Republic and the U.S., while inconsistent parenting produced higher odds for feeling lonely among all groups of adolescents except Czech females. Our findings accord with those from previous studies which have linked parental warmth [14–16] and inconsistent parenting [13] to adolescent loneliness, but do not agree with the earlier finding that parental involvement decreases the risk of adolescent loneliness [17]. There are several ways in which parenting might be associated with adolescent loneliness. Parental warmth is one of the main elements of what has been termed ‘authoritative parenting’ which has been linked to the development of adolescent competence [41], with warmth associated with more sociable behaviour in children [14], and possibly, the degree to which they try to establish relations with other people [42]. Parenting styles might also provide models from which adolescents learn interactional skills [42]. If this is correct, then inconsistent parenting might be especially detrimental. Moreover, parents’ unpredictable behaviour has previously been linked to children’s possible social withdrawal [42] with the implications this carries for future loneliness.

Having at least one close friend was a protective factor against loneliness among U.S. boys and Czech girls. This might be explained by the important role that close friends perform in terms of providing many different types of support during adolescence [8]. Peer support has also been linked to the development of adolescent self-esteem [43]. This might be important as low levels of self-esteem have been previously reported to be a risk factor for adolescent loneliness [44, 45]. It is also possible that the differences we observed between close friendship and loneliness across the countries might be related to differences in the nature of friendship across these societies. Earlier research has shown for example, that compared to Americans, Russians tend to have a lower number of friends with whom they share less personal information [46]. This might help explain why an absence of close friends was not associated with loneliness among Russian girls or boys.

The findings from the present study also highlight the important role of personal characteristics in adolescent loneliness, as the highest odds for feeling lonely in boys and girls across the countries were reported by those adolescents, who answered that it was ‘Certainly true’ that they were shy. This relation between shyness and loneliness has been observed in a number of previous studies of adolescent loneliness [45, 47, 48]. It has been suggested that shyness may be causally related to the development of feelings of loneliness [49], possibly because being shy results in adolescents avoiding social situations [47] which inhibits the formation of social relations [50]. This avoidance might emanate from the greater anxiety and/or poor social skills that shy people display [49] in social situations.

The fact that peer victimisation was linked to adolescent loneliness across the three countries also accords with the results from previous Western studies [20, 38]. Victimisation may be linked to loneliness through the effects it has on friendship formation as previous research has shown that victimised children have more problems with forming new friendships [51]. The fact that victimisation was associated with female loneliness in all of the countries but was only linked to feeling lonely among U.S. boys also provides support for the notion that peer victimisation might have different outcomes for girls and boys. Specifically, it has been suggested that for boys, victimisation might result in an increased risk of future victimisation (after leaving the peer group), whereas for girls it has been associated with greater social avoidance [52]. If social avoidance is one outcome of female victimisation, it might explain why victimised girls were at an increased risk of feeling lonely in each of our study countries.

### Loneliness and adolescent health

Loneliness was associated with depressive, anxiety and somatic symptoms among boys and girls in all of the countries. Although there have been several previous studies on the association between loneliness and psychological health problems among children, the relation between loneliness and somatic symptoms has been little studied to date [24]. Our findings accord with those from recent studies that have linked adolescent loneliness to worse mental health [53] including depressive [34] and anxiety symptoms [54], and with studies that have shown an association between loneliness and various somatic symptoms [25] such as headaches [24]. An earlier study found that the relation between loneliness and somatic symptoms was seen only among boys [25], while our results suggest that this association is seen among both sexes but that boys report a greater number of symptoms. It is uncertain which mechanisms underlie the relation between loneliness and poorer health. Cacioppo and Patrick [55] have suggested that there may be as many as five intersecting causal pathways that link loneliness and poorer health including worse health behaviours, the perception of everyday stressors as being more severe and poorer quality sleep. In terms of the current study, it is possible that poorer health behaviours might be central to this association in adolescence. A recent study showed that adolescent loneliness is linked to different forms of substance use in some of these countries [36], while previous research has demonstrated an association between adolescent alcohol and drug use and somatic symptoms [56] and psychological ill health [57, 58].

### Study limitations

The findings of this study should be considered in light of several limitations. First, we used a single-item study question to measure loneliness. This may have been problematic as recent research has indicated that single-item questions and multiple-item scales can produce different results in terms of the prevalence of loneliness and the characteristics associated with it [59]. Other research has however, suggested that the single-item measure of loneliness is valid and continues to be widely used when survey data is being collected [38], possibly because it enables quicker responses. Second, we had to rely on self-reports for the variables used in this study without being able to check the accuracy of this information. It is possible that this might have resulted in bias if for example, there were systematic differences in self-disclosure across the lonely and non-lonely groups. In particular, it has been suggested that as lonely children generally tend to have more negative opinions about other people, they may be prone to evaluate parental behaviour in a less positive manner (which has been termed the ‘negative perception’ hypothesis) [17]. While this cannot be discounted as possibly having occurred in the current study, the fact that we obtained different results for the three parental behaviour variables suggests that responses were not being driven solely by a negative mind set. Third, we lacked information about certain variables which have been previously shown to be important when it comes to understanding adolescent loneliness e.g. self-esteem [39]. Fourth, we examined a measure of overall loneliness. A recent study, has indicated however, that adolescent loneliness not only occurs across different spheres (family, peer and in romantic contexts), but that these different sources of loneliness are related to psychopathology (e.g. anxiety) in different ways [54]. This suggests that future studies should employ more nuanced definitions of loneliness when examining its effects on adolescent well-being. Fifth, the parenting scale had not been formally validated and the alpha value for inconsistent parenting was low (α = 0.65). Sixth, the odds ratios and confidence intervals for depressive symptoms in the multivariable analysis were large. Further investigation showed that this was not because of multicolinearity. Nonetheless, these results should be interpreted with caution. Finally, as this study was cross-sectional causality could not be determined.

## Conclusion

This study has shown that a variety of factors are associated with loneliness during adolescence and that lonely adolescents have elevated odds for experiencing poorer psychological and somatic health across countries that differ historically and culturally. This highlights the necessity of efforts to address loneliness and its negative effects among young people. Although there are several forms of intervention that have been previously directed against loneliness [60], as yet, there has been comparatively little research on ways to protect against adolescent loneliness and its effects in arenas such as schools [61]. Given the importance of factors such as shyness, parenting and victimisation for loneliness across locations, the results from the present study suggest that future interventions against loneliness should occur in different settings (e.g. in the home and school) that are associated with adolescent loneliness.

### Ethics approval and consent to participate

Ethical approval for the study was obtained from ethical committees at the Northern State Medical University in Arkhangelsk, Yale University School of Medicine and the Institute of Psychology, Academy of Sciences of the Czech Republic, v.v.i. While planning the study we used as a standard the procedure of passive informed consent approved by the ethical committee at Yale University where the survey instrument was developed. Hence, no written informed consent was obtained from parents/guardians but they had the possibility to refuse their child’s participation while the children themselves signed an informed consent form.

### Consent for publication

Not applicable.

### Availability of data and materials

The data for the Russian and Czech samples are available from the authors upon request. The data from the U.S. sample cannot be shared because of the data storage requirements and data availability limitations specified by the Yale Institutional Review Board. The survey questions that were included in this study have been uploaded as a supplementary file (see Additional file 1).

## Additional file

Additional file 1: Survey questions. (DOCX 20 kb)

## Abbreviations

CES-D: Centre for Epidemiologic Studies-Depression Scale
SAHA: Social and Health Assessment
