# Supplementary material for: Loneliness and its association with psychological and somatic health problems among Czech, Russian and U.S. adolescents
Source: BMC Psychiatry. 2016 May 4;16:128. doi: 10.1186/s12888-016-0829-2 (PMC4857285; doi:10.1186/s12888-016-0829-2)
Supplement: Additional file 1: — Survey questions. (DOCX 20 kb) [file 12888_2016_829_MOESM1_ESM.docx]

**Additional File 1 – Survey Questions**

Loneliness

Think about how you felt or behaved in the **past 30 days**.

Not true Somewhat True Certainly True

I felt lonely 1 2 3

Personal characteristics

Please circle if the following statements are Not True, Somewhat True, or Certainly True for you.

Not true Somewhat True Certainly True

I am shy 1 2 3

Friendship Ties

How many close friends do you have? 0 1 2 3-4 5 or more

Parental education

How far did your father or male guardian go in school?

| Some grade school or high school | Graduated from high school | Some college or training after high school | Graduated from college | Don’t know |
| --- | --- | --- | --- | --- |

How far did your mother or female guardian go in school?

| Some grade school or high school | Graduated from high school | Some college or training after high school | Graduated from college | Don’t know |
| --- | --- | --- | --- | --- |

Household size

**Think about the home you live in most of the time.**

How many people are living in this home now including yourself?

| 1 | 2 | 3 | 4 | 5 | 6 | 7 | 8 | 9 | 10 or more |
| --- | --- | --- | --- | --- | --- | --- | --- | --- | --- |

Family structure

Circle below who those adults are.

| Mother | Father | Grandmother | Grandfather | Foster mother | Foster farther |
| --- | --- | --- | --- | --- | --- |
| Stepmother | Stepfather | Aunt | Uncle | Other female adults | Other male adults |

Parenting

**My parents…**

|  | Never | Rarely | Sometimes | Often |
| --- | --- | --- | --- | --- |
| Forget a rule they have made | 1 | 2 | 3 | 4 |
| Nag me about little things | 1 | 2 | 3 | 4 |
| Only keep rules when it suits them | 1 | 2 | 3 | 4 |
| Threaten punishment more often than they use it | 1 | 2 | 3 | 4 |
| Enforce a rule or do not enforce a rule depending upon their mood | 1 | 2 | 3 | 4 |
|  |  |  |  |  |
| Ask me about my life | 1 | 2 | 3 | 4 |
| Spend time on activities at my school | 1 | 2 | 3 | 4 |
| Spend their free time with me | 1 | 2 | 3 | 4 |
| Encourage me to be interested in different things | 1 | 2 | 3 | 4 |
| Give me good advice | 1 | 2 | 3 | 4 |
| Are interested in my friends | 1 | 2 | 3 | 4 |
|  |  |  |  |  |
| Are kind to me | 1 | 2 | 3 | 4 |
| Hug or kiss me | 1 | 2 | 3 | 4 |
| Are proud of me | 1 | 2 | 3 | 4 |
| Show their love for me | 1 | 2 | 3 | 4 |
| Make me feel good when I am with them | 1 | 2 | 3 | 4 |

School attachment

Please circle if the following statements are Definitely Not True, Mostly Not True, Mostly True, or Definitely True for you

|  | Definitely Not True | Mostly Not True | Mostly True | Definitely True |
| --- | --- | --- | --- | --- |
| I like school | 1 | 2 | 3 | 4 |

Peer victimisation

During this school year other kids **in school…**

|  | Not at All | Once | 2-3 Times | 4 or More Times |
| --- | --- | --- | --- | --- |
| Called me names or swore at me | 0 | 1 | 2 | 3 |
| Tried to get me into trouble with my friends | 0 | 1 | 2 | 3 |
| Took something without permission or stole things from me | 0 | 1 | 2 | 3 |
| Made fun of me for some reason | 0 | 1 | 2 | 3 |
| Made me feel uncomfortable by standing too close or touching me | 0 | 1 | 2 | 3 |
| Punched, kicked or beat me up | 0 | 1 | 2 | 3 |
| Hurt me physically in some way | 0 | 1 | 2 | 3 |
| Tried to break or damage something of mine | 0 | 1 | 2 | 3 |
| Refused to talk to me or made other people not talk to me | 0 | 1 | 2 | 3 |

Depressive symptoms

Think about how you have felt or behaved in the **past 30 days.**

|  | Not True | Somewhat True | Certainly True |
| --- | --- | --- | --- |
| I did not feel like eating; my appetite was poor | 0 | 1 | 2 |
| I felt that I could not shake off my sad feelings even with help from my family or friends | 0 | 1 | 2 |
| I felt like crying | 0 | 1 | 2 |
| I felt really down | 0 | 1 | 2 |
| I felt that many bad things were my fault | 0 | 1 | 2 |
| I was tired | 0 | 1 | 2 |
| I have lost my interest in other people or things | 0 | 1 | 2 |
| I did not like myself | 0 | 1 | 2 |
| I felt bothered by people and things | 0 | 1 | 2 |

Anxiety symptoms

Please circle if the following statements are Not True, Somewhat True, or Certainly True for you.

|  | Not True | Somewhat True | Certainly True |
| --- | --- | --- | --- |
| I worry about other people liking me | 0 | 1 | 2 |
| I feel nervous when I get called on in class | 0 | 1 | 2 |
| I worry about being as good as other kids | 0 | 1 | 2 |
| I worry about what others think about me | 0 | 1 | 2 |
| I worry about what is going to happen in the future | 0 | 1 | 2 |
| I worry about how well I do things | 0 | 1 | 2 |
| I feel nervous if I have to do something in front of a group of people | 0 | 1 | 2 |
| I stay away from things that make me nervous | 0 | 1 | 2 |
| I worry about doing something stupid or embarrassing | 0 | 1 | 2 |
| I avoid going to unfamiliar places | 0 | 1 | 2 |
| I worry about things that I have done | 0 | 1 | 2 |
| I feel nervous with people I don’t know well | 0 | 1 | 2 |

Somatic symptoms

**These next questions are about how you’ve been feeling lately**

During the **past 30 days…**

|  | Not True | Somewhat True | Certainly True |
| --- | --- | --- | --- |
| I had headaches | 0 | 1 | 2 |
| I had stomach aches | 0 | 1 | 2 |
| I had aches or pains | 0 | 1 | 2 |
| I had nausea | 0 | 1 | 2 |
| I had problems with my eyes | 0 | 1 | 2 |
| I had rashes or other skin problems | 0 | 1 | 2 |
| I was vomiting | 0 | 1 | 2 |
